# 3-(2*H*-1,3-Benzodioxol-5-ylmeth­yl)-2-(2-meth­oxy­phen­yl)-1,3-thia­zolidin-4-one

**DOI:** 10.1107/S1600536811041262

**Published:** 2011-10-22

**Authors:** Victor Facchinetti, Claudia R. B. Gomes, Wilson Cunico, Solange M. S. V. Wardell, James L. Wardell, Edward R. T. Tiekink

**Affiliations:** a Fundação Oswaldo Cruz, Instituto de Tecnologia em Fármacos–Farmanguinhos, R. Sizenando Nabuco 100, Manguinhos, 21041-250 Rio de Janeiro, RJ, Brazil; b Departamento de Química Orgânica, Universidade Federal de Pelotas (UFPel), Campus Universitário, s/n, Caixa Postal 354, 96010-900 Pelotas, RS, Brazil; c CHEMSOL, 1 Harcourt Road, Aberdeen AB15 5NY, Scotland; d Centro de Desenvolvimento Tecnológico em Saúde (CDTS), Fundação Oswaldo Cruz (FIOCRUZ), Casa Amarela, Campus de Manguinhos, Av. Brasil 4365, 21040-900, Rio de Janeiro, RJ, Brazil; e Department of Chemistry, University of Malaya, 50603 Kuala Lumpur, Malaysia

## Abstract

The title mol­ecule, C_18_H_17_NO_4_S, features a 1,3-thia­zolidine ring that is twisted about the S—C(methyl­ene) bond. With reference to this ring, the 1,3-benzodioxole and benzene rings lie to either side and form dihedral angles of 69.72 (16) and 83.60 (14)°, respectively, with the central ring. Significant twisting in the mol­ecule is confirmed by the dihedral angle of 79.91 (13)° formed between the outer rings. Linear supra­molecular chains along the *a*-axis direction mediated by C—H⋯O inter­actions feature in the crystal packing.

## Related literature

For background to the biological activity of thia­zolidinones, see: Cunico *et al.* (2008*a* ▶); Solomon *et al.* (2007 ▶); Kavitha *et al.* (2006 ▶); Sharma *et al.* (2006 ▶); Ravichandran *et al.* (2009 ▶); Rao *et al.* (2004 ▶). For background to the synthesis, see: Cunico *et al.* (2008*b* ▶); Rawal *et al.* (2008 ▶), Gomes *et al.* (2010 ▶), Neuenfeldt *et al.* (2011 ▶). For related studies on the synthesis and biological evaluation of thia­zolidinones, see: Cunico *et al.* (2006 ▶, 2007 ▶). For a thia­zolidinone structure, see: Neuenfeldt *et al.* (2009 ▶).
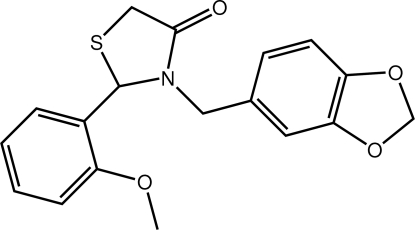


## Experimental

### 

#### Crystal data

C_18_H_17_NO_4_S
*M* *_r_* = 343.39
Monoclinic, 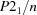

*a* = 6.8137 (3) Å
*b* = 12.5753 (7) Å
*c* = 18.5071 (9) Å
β = 91.825 (3)°
*V* = 1584.96 (14) Å^3^
*Z* = 4
Mo *K*α radiation
μ = 0.23 mm^−1^
*T* = 120 K
0.16 × 0.06 × 0.05 mm

#### Data collection

Bruker–Nonius APEXII CCD camera on κ-goniostat diffractometer
Absorption correction: multi-scan (*SADABS*; Sheldrick, 2007 ▶) *T*_min_ = 0.553, *T*_max_ = 0.746
21683 measured reflections
3625 independent reflections
1935 reflections with *I* > 2σ(*I*)
*R*_int_ = 0.159

#### Refinement

*R*[*F* ^2^ > 2σ(*F* ^2^)] = 0.068
*wR*(*F* ^2^) = 0.180
*S* = 1.02
3625 reflections
218 parameters
H-atom parameters constrained
Δρ_max_ = 0.33 e Å^−3^
Δρ_min_ = −0.43 e Å^−3^

### 

Data collection: *COLLECT* (Hooft, 1998 ▶); cell refinement: *DENZO* (Otwinowski & Minor, 1997 ▶) and *COLLECT*; data reduction: *DENZO* and *COLLECT*; program(s) used to solve structure: *SHELXS97* (Sheldrick, 2008 ▶); program(s) used to refine structure: *SHELXL97* (Sheldrick, 2008 ▶); molecular graphics: *ORTEP-3* (Farrugia, 1997 ▶) and *DIAMOND* (Brandenburg, 2006 ▶); software used to prepare material for publication: *publCIF* (Westrip, 2010 ▶).

## Supplementary Material

Crystal structure: contains datablock(s) global, I. DOI: 10.1107/S1600536811041262/hb6438sup1.cif

Structure factors: contains datablock(s) I. DOI: 10.1107/S1600536811041262/hb6438Isup2.hkl

Supplementary material file. DOI: 10.1107/S1600536811041262/hb6438Isup3.cml

Additional supplementary materials:  crystallographic information; 3D view; checkCIF report

## Figures and Tables

**Table 1:** Hydrogen-bond geometry (Å, °)

*D*—H⋯*A*	*D*—H	H⋯*A*	*D*⋯*A*	*D*—H⋯*A*
C9—H9⋯O1^i^	0.95	2.36	3.302 (4)	170
C13—H13⋯O1^ii^	0.95	2.43	3.352 (4)	163

Symmetry codes: (i) 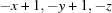; (ii) 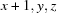.

**Fig. 1.**
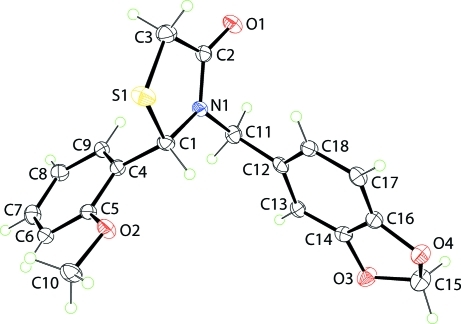
The molecular structure of (I) showing displacement ellipsoids at the 50% probability level.

**Fig. 2.**
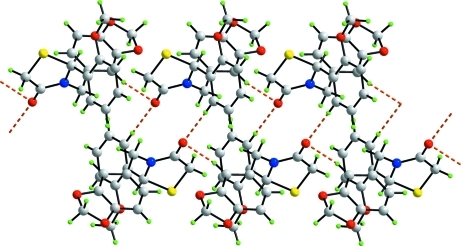
A view of the linear supramolecular chain propogated down the a axis via C—H···O interactions (orange dashed lines) in the crystal structure of (I).

## supplementary crystallographic information

### Comment

Thiazolidinones constitute an important group of heterocyclic compounds (Cunico *et al.*, 2008*a*), having valuable biological uses, for example, as anti-malarial (Solomon *et al.*, 2007), anti-microbial (Kavitha *et al.*, 2006), anti-inflammatory (Sharma *et al.*, 2006), and anti-viral agents, especially as anti-HIV agents (Ravichandran *et al.*, 2009; Rao *et al.*, 2004). The main synthetic routes to 1,3-thiazolidin-4-ones involve three components (an aldehyde, an amine and mercaptoacetic acid), either in a one- or two-step process (Cunico *et al.*, 2008*a*; Rawal *et al.*, 2008), and also under ultrasound irradiation (Neuenfeldt *et al.*, 2011). The structure of 1-thia-4-azaspiro[4.5]decan-3-one has been reported recently (Neuenfeldt *et al.*, 2009). In continuation of our research on thiazolidinones, (Cunico *et al.*, 2006; Cunico *et al.*, 2007; Cunico *et al.*, 2008*b*; Gomes *et al.*, 2010; Neuenfeldt *et al.*, 2011), we now wish to report the structure of 2-(2-methoxybenzaldehyde)-3-piperonyl-1,3-thiazolidin-4-one, (I), synthesized, as reported from piperonylamine, 2-methoxybenzaldehyde and mercaptoacetic acid under ultrasound irradiation (Neuenfeldt *et al.*, 2011). The sample used in the structure determination was grown from its EtOH solution.

The thiazolidinyl ring in (I), Fig. 1, is twisted about the S1—C3 bond but, the deviations from co-planarity for the five atoms are not great, *i.e*. the maximum and minimum deviations are 0.109 (1) Å for atom S1 and -0.117 (4) Å for atom C3; the ketone-O1 atom lies 0.244 (2) Å out of the least-squares plane through the five-membered ring. The dioxole ring has an envelope conformation with the C15 atom being the flap atom. The r.m.s. deviation for the 13 non-hydrogen atoms comprising the 1,3-benzodioxole ring is 0.110 Å. With reference to the thiazolidinyl ring, the 1,3-benzodioxole and benzene rings lie to either side and form dihedral angles with this ring of 69.72 (16) and 83.60 (14)°, respectively. The outer rings form a dihedral angle of 79.91 (13)° with each other, indicating that the molecule is highly twisted.

The most prominent feature of the crystal packing is the formation of C—H···O interactions involving the bifurcated carbonyl-O1 atom, Table 1. These lead to linear supramolecular chains along the *a* axis, Fig. 2.

### Experimental

The title compound was synthesized as described in the literature (Neuenfeldt *et al.*, 2011) and crystals were obtained from its EtOH solution.

### Refinement

The C-bound H atoms were geometrically placed (C—H = 0.95–1.00 Å) and refined as riding with *U_iso_*(H) = 1.2*U_eq_*(C).

### Crystal data

**Table:**

C_18_H_17_NO_4_S	*F*(000) = 720
*M**_r_* = 343.39	*D*_x_ = 1.439 Mg m^−^^3^
Monoclinic, *P*2_1_/*n*	Mo *K*α radiation, λ = 0.71073 Å
Hall symbol: -P 2yn	Cell parameters from 7149 reflections
*a* = 6.8137 (3) Å	θ = 2.9–27.5°
*b* = 12.5753 (7) Å	µ = 0.23 mm^−^^1^
*c* = 18.5071 (9) Å	*T* = 120 K
β = 91.825 (3)°	Block, colourless
*V* = 1584.96 (14) Å^3^	0.16 × 0.06 × 0.05 mm
*Z* = 4	

### Data collection

**Table:**

Bruker–Nonius APEXII CCD camera on κ-goniostat diffractometer	3625 independent reflections
Radiation source: Bruker–Nonius FR591 rotating anode	1935 reflections with *I* > 2σ(*I*)
10cm confocal mirrors	*R*_int_ = 0.159
Detector resolution: 9.091 pixels mm^-1^	θ_max_ = 27.5°, θ_min_ = 3.2°
φ and ω scans	*h* = −8→8
Absorption correction: multi-scan (*SADABS*; Sheldrick, 2007)	*k* = −15→16
*T*_min_ = 0.553, *T*_max_ = 0.746	*l* = −23→24
21683 measured reflections	

### Refinement

**Table:**

Refinement on *F*^2^	Primary atom site location: structure-invariant direct methods
Least-squares matrix: full	Secondary atom site location: difference Fourier map
*R*[*F*^2^ > 2σ(*F*^2^)] = 0.068	Hydrogen site location: inferred from neighbouring sites
*wR*(*F*^2^) = 0.180	H-atom parameters constrained
*S* = 1.02	*w* = 1/[σ^2^(*F*_o_^2^) + (0.0754*P*)^2^] where *P* = (*F*_o_^2^ + 2*F*_c_^2^)/3
3625 reflections	(Δ/σ)_max_ < 0.001
218 parameters	Δρ_max_ = 0.33 e Å^−^^3^
0 restraints	Δρ_min_ = −0.43 e Å^−^^3^

### Special details

**Table:**

Geometry. All s.u.'s (except the s.u. in the dihedral angle between two l.s. planes) are estimated using the full covariance matrix. The cell s.u.'s are taken into account individually in the estimation of s.u.'s in distances, angles and torsion angles; correlations between s.u.'s in cell parameters are only used when they are defined by crystal symmetry. An approximate (isotropic) treatment of cell s.u.'s is used for estimating s.u.'s involving l.s. planes.
Refinement. Refinement of *F*^2^ against ALL reflections. The weighted *R*-factor *wR* and goodness of fit *S* are based on *F*^2^, conventional *R*-factors *R* are based on *F*, with *F* set to zero for negative *F*^2^. The threshold expression of *F*^2^ > 2σ(*F*^2^) is used only for calculating *R*-factors(gt) *etc*. and is not relevant to the choice of reflections for refinement. *R*-factors based on *F*^2^ are statistically about twice as large as those based on *F*, and *R*- factors based on ALL data will be even larger.

### Fractional atomic coordinates and isotropic or equivalent isotropic displacement parameters (Å^2^)

**Table:**

	*x*	*y*	*z*	*U*_iso_*/*U*_eq_	
S1	0.48794 (13)	0.72256 (9)	0.17227 (5)	0.0365 (3)	
O1	0.3856 (3)	0.60647 (19)	−0.01787 (12)	0.0321 (6)	
O2	0.9136 (3)	0.76552 (19)	0.24212 (12)	0.0291 (6)	
O3	1.2380 (3)	0.9163 (2)	−0.11437 (14)	0.0358 (6)	
O4	1.0102 (3)	1.05303 (19)	−0.11655 (13)	0.0328 (6)	
N1	0.6500 (4)	0.6556 (2)	0.05355 (13)	0.0231 (6)	
C1	0.7138 (4)	0.6948 (3)	0.12420 (17)	0.0246 (8)	
H1	0.7849	0.7635	0.1174	0.029*	
C2	0.4558 (5)	0.6343 (3)	0.04091 (18)	0.0260 (8)	
C3	0.3391 (5)	0.6492 (3)	0.10759 (19)	0.0358 (9)	
H3A	0.3034	0.5792	0.1279	0.043*	
H3B	0.2167	0.6888	0.0957	0.043*	
C4	0.8488 (4)	0.6199 (3)	0.16581 (17)	0.0239 (8)	
C5	0.9521 (4)	0.6609 (3)	0.22695 (17)	0.0236 (8)	
C6	1.0804 (5)	0.5969 (3)	0.26668 (18)	0.0278 (8)	
H6	1.1525	0.6253	0.3070	0.033*	
C7	1.1036 (5)	0.4905 (3)	0.24740 (18)	0.0300 (9)	
H7	1.1917	0.4465	0.2747	0.036*	
C8	0.9991 (5)	0.4487 (3)	0.18869 (18)	0.0289 (8)	
H8	1.0127	0.3757	0.1765	0.035*	
C9	0.8740 (5)	0.5139 (3)	0.14744 (17)	0.0260 (8)	
H9	0.8053	0.4856	0.1063	0.031*	
C10	0.9967 (5)	0.8064 (3)	0.30861 (18)	0.0321 (9)	
H10A	0.9499	0.7642	0.3491	0.048*	
H10B	0.9568	0.8807	0.3145	0.048*	
H10C	1.1402	0.8022	0.3077	0.048*	
C11	0.7881 (5)	0.6555 (3)	−0.00479 (17)	0.0281 (8)	
H11A	0.7309	0.6146	−0.0460	0.034*	
H11B	0.9099	0.6188	0.0119	0.034*	
C12	0.8399 (5)	0.7660 (3)	−0.03050 (16)	0.0254 (8)	
C13	1.0282 (5)	0.7820 (3)	−0.05777 (17)	0.0245 (8)	
H13	1.1244	0.7273	−0.0570	0.029*	
C14	1.0652 (4)	0.8808 (3)	−0.08553 (18)	0.0264 (8)	
C15	1.1839 (5)	1.0137 (3)	−0.1505 (2)	0.0372 (9)	
H15A	1.1562	1.0004	−0.2026	0.045*	
H15B	1.2917	1.0662	−0.1456	0.045*	
C16	0.9303 (5)	0.9626 (3)	−0.08612 (18)	0.0285 (8)	
C17	0.7480 (5)	0.9499 (3)	−0.05749 (17)	0.0280 (8)	
H17	0.6563	1.0068	−0.0562	0.034*	
C18	0.7038 (5)	0.8489 (3)	−0.03018 (17)	0.0260 (8)	
H18	0.5781	0.8366	−0.0110	0.031*	

### Atomic displacement parameters (Å^2^)

**Table:**

	*U*^11^	*U*^22^	*U*^33^	*U*^12^	*U*^13^	*U*^23^
S1	0.0270 (5)	0.0496 (7)	0.0329 (5)	0.0028 (4)	0.0009 (4)	−0.0113 (5)
O1	0.0314 (13)	0.0343 (16)	0.0300 (14)	0.0017 (11)	−0.0083 (11)	−0.0041 (12)
O2	0.0332 (13)	0.0292 (15)	0.0245 (13)	0.0021 (11)	−0.0070 (10)	−0.0028 (11)
O3	0.0291 (13)	0.0316 (16)	0.0472 (16)	0.0008 (11)	0.0082 (11)	0.0041 (12)
O4	0.0329 (14)	0.0290 (15)	0.0367 (14)	−0.0010 (11)	0.0049 (11)	0.0016 (12)
N1	0.0231 (14)	0.0276 (17)	0.0183 (13)	−0.0027 (12)	−0.0017 (11)	−0.0009 (12)
C1	0.0246 (17)	0.026 (2)	0.0232 (17)	−0.0041 (14)	−0.0026 (14)	−0.0039 (15)
C2	0.0269 (17)	0.023 (2)	0.0279 (19)	−0.0020 (14)	−0.0041 (15)	0.0032 (16)
C3	0.0270 (18)	0.050 (3)	0.0305 (19)	−0.0054 (17)	0.0024 (15)	−0.0001 (18)
C4	0.0199 (16)	0.030 (2)	0.0223 (17)	−0.0005 (14)	0.0019 (13)	0.0035 (15)
C5	0.0213 (16)	0.028 (2)	0.0218 (17)	−0.0039 (14)	0.0000 (13)	−0.0007 (15)
C6	0.0255 (17)	0.035 (2)	0.0225 (18)	−0.0062 (16)	−0.0016 (14)	0.0015 (16)
C7	0.0307 (19)	0.029 (2)	0.030 (2)	0.0074 (16)	0.0020 (16)	0.0040 (17)
C8	0.0306 (19)	0.027 (2)	0.0294 (19)	−0.0010 (15)	0.0006 (15)	−0.0041 (16)
C9	0.0280 (18)	0.029 (2)	0.0207 (17)	−0.0037 (15)	−0.0012 (14)	0.0010 (15)
C10	0.037 (2)	0.031 (2)	0.0284 (19)	−0.0037 (17)	−0.0062 (15)	−0.0091 (17)
C11	0.0308 (18)	0.031 (2)	0.0220 (17)	0.0019 (16)	0.0018 (14)	−0.0015 (16)
C12	0.0283 (18)	0.031 (2)	0.0164 (17)	−0.0006 (15)	−0.0049 (14)	−0.0026 (15)
C13	0.0251 (17)	0.024 (2)	0.0239 (17)	0.0047 (14)	−0.0016 (14)	0.0000 (15)
C14	0.0231 (17)	0.031 (2)	0.0250 (18)	−0.0010 (15)	−0.0005 (14)	−0.0027 (16)
C15	0.036 (2)	0.032 (2)	0.044 (2)	0.0046 (17)	0.0117 (18)	0.0017 (18)
C16	0.0322 (19)	0.029 (2)	0.0240 (18)	−0.0043 (16)	−0.0032 (15)	0.0000 (16)
C17	0.0296 (19)	0.030 (2)	0.0239 (18)	0.0047 (15)	−0.0020 (15)	−0.0021 (16)
C18	0.0225 (16)	0.030 (2)	0.0256 (17)	−0.0013 (15)	−0.0020 (14)	0.0000 (16)

### Geometric parameters (Å, °)

**Table:**

S1—C3	1.799 (4)	C7—C8	1.384 (5)
S1—C1	1.836 (3)	C7—H7	0.9500
O1—C2	1.225 (4)	C8—C9	1.393 (5)
O2—C5	1.372 (4)	C8—H8	0.9500
O2—C10	1.433 (4)	C9—H9	0.9500
O3—C14	1.382 (4)	C10—H10A	0.9800
O3—C15	1.437 (4)	C10—H10B	0.9800
O4—C16	1.388 (4)	C10—H10C	0.9800
O4—C15	1.445 (4)	C11—C12	1.514 (5)
N1—C2	1.363 (4)	C11—H11A	0.9900
N1—C1	1.451 (4)	C11—H11B	0.9900
N1—C11	1.455 (4)	C12—C18	1.395 (5)
C1—C4	1.510 (5)	C12—C13	1.408 (5)
C1—H1	1.0000	C13—C14	1.372 (5)
C2—C3	1.501 (5)	C13—H13	0.9500
C3—H3A	0.9900	C14—C16	1.379 (5)
C3—H3B	0.9900	C15—H15A	0.9900
C4—C9	1.387 (5)	C15—H15B	0.9900
C4—C5	1.411 (4)	C16—C17	1.375 (5)
C5—C6	1.382 (5)	C17—C18	1.404 (5)
C6—C7	1.395 (5)	C17—H17	0.9500
C6—H6	0.9500	C18—H18	0.9500
			
C3—S1—C1	92.50 (15)	C4—C9—H9	119.7
C5—O2—C10	116.5 (3)	C8—C9—H9	119.7
C14—O3—C15	104.2 (2)	O2—C10—H10A	109.5
C16—O4—C15	103.5 (3)	O2—C10—H10B	109.5
C2—N1—C1	118.9 (3)	H10A—C10—H10B	109.5
C2—N1—C11	121.3 (3)	O2—C10—H10C	109.5
C1—N1—C11	119.1 (3)	H10A—C10—H10C	109.5
N1—C1—C4	114.1 (3)	H10B—C10—H10C	109.5
N1—C1—S1	105.6 (2)	N1—C11—C12	113.3 (3)
C4—C1—S1	112.2 (2)	N1—C11—H11A	108.9
N1—C1—H1	108.2	C12—C11—H11A	108.9
C4—C1—H1	108.2	N1—C11—H11B	108.9
S1—C1—H1	108.2	C12—C11—H11B	108.9
O1—C2—N1	123.9 (3)	H11A—C11—H11B	107.7
O1—C2—C3	124.3 (3)	C18—C12—C13	120.5 (3)
N1—C2—C3	111.8 (3)	C18—C12—C11	121.5 (3)
C2—C3—S1	108.0 (2)	C13—C12—C11	117.9 (3)
C2—C3—H3A	110.1	C14—C13—C12	116.4 (3)
S1—C3—H3A	110.1	C14—C13—H13	121.8
C2—C3—H3B	110.1	C12—C13—H13	121.8
S1—C3—H3B	110.1	C13—C14—C16	123.2 (3)
H3A—C3—H3B	108.4	C13—C14—O3	127.3 (3)
C9—C4—C5	119.0 (3)	C16—C14—O3	109.4 (3)
C9—C4—C1	123.5 (3)	O3—C15—O4	106.9 (3)
C5—C4—C1	117.5 (3)	O3—C15—H15A	110.3
O2—C5—C6	124.8 (3)	O4—C15—H15A	110.3
O2—C5—C4	114.9 (3)	O3—C15—H15B	110.3
C6—C5—C4	120.3 (3)	O4—C15—H15B	110.3
C5—C6—C7	119.8 (3)	H15A—C15—H15B	108.6
C5—C6—H6	120.1	C17—C16—C14	121.4 (3)
C7—C6—H6	120.1	C17—C16—O4	128.5 (3)
C8—C7—C6	120.4 (3)	C14—C16—O4	110.1 (3)
C8—C7—H7	119.8	C16—C17—C18	116.8 (3)
C6—C7—H7	119.8	C16—C17—H17	121.6
C7—C8—C9	119.8 (3)	C18—C17—H17	121.6
C7—C8—H8	120.1	C12—C18—C17	121.7 (3)
C9—C8—H8	120.1	C12—C18—H18	119.2
C4—C9—C8	120.7 (3)	C17—C18—H18	119.2
			
C2—N1—C1—C4	115.2 (3)	C5—C4—C9—C8	0.1 (5)
C11—N1—C1—C4	−74.1 (4)	C1—C4—C9—C8	−179.2 (3)
C2—N1—C1—S1	−8.5 (4)	C7—C8—C9—C4	−1.8 (5)
C11—N1—C1—S1	162.2 (2)	C2—N1—C11—C12	101.4 (4)
C3—S1—C1—N1	14.3 (2)	C1—N1—C11—C12	−69.0 (4)
C3—S1—C1—C4	−110.5 (3)	N1—C11—C12—C18	−33.1 (4)
C1—N1—C2—O1	175.9 (3)	N1—C11—C12—C13	149.5 (3)
C11—N1—C2—O1	5.4 (5)	C18—C12—C13—C14	−2.1 (5)
C1—N1—C2—C3	−4.0 (4)	C11—C12—C13—C14	175.3 (3)
C11—N1—C2—C3	−174.5 (3)	C12—C13—C14—C16	1.4 (5)
O1—C2—C3—S1	−165.0 (3)	C12—C13—C14—O3	179.1 (3)
N1—C2—C3—S1	14.9 (4)	C15—O3—C14—C13	166.6 (3)
C1—S1—C3—C2	−16.7 (3)	C15—O3—C14—C16	−15.4 (4)
N1—C1—C4—C9	−14.6 (4)	C14—O3—C15—O4	23.8 (4)
S1—C1—C4—C9	105.4 (3)	C16—O4—C15—O3	−23.0 (3)
N1—C1—C4—C5	166.0 (3)	C13—C14—C16—C17	0.9 (5)
S1—C1—C4—C5	−73.9 (3)	O3—C14—C16—C17	−177.2 (3)
C10—O2—C5—C6	−7.3 (4)	C13—C14—C16—O4	179.2 (3)
C10—O2—C5—C4	172.6 (3)	O3—C14—C16—O4	1.1 (4)
C9—C4—C5—O2	−178.2 (3)	C15—O4—C16—C17	−168.2 (3)
C1—C4—C5—O2	1.2 (4)	C15—O4—C16—C14	13.7 (4)
C9—C4—C5—C6	1.7 (4)	C14—C16—C17—C18	−2.3 (5)
C1—C4—C5—C6	−179.0 (3)	O4—C16—C17—C18	179.8 (3)
O2—C5—C6—C7	178.1 (3)	C13—C12—C18—C17	0.8 (5)
C4—C5—C6—C7	−1.8 (5)	C11—C12—C18—C17	−176.6 (3)
C5—C6—C7—C8	0.0 (5)	C16—C17—C18—C12	1.5 (5)
C6—C7—C8—C9	1.7 (5)		

### Hydrogen-bond geometry (Å, °)

**Table:**

*D*—H···*A*	*D*—H	H···*A*	*D*···*A*	*D*—H···*A*
C9—H9···O1^i^	0.95	2.36	3.302 (4)	170
C13—H13···O1^ii^	0.95	2.43	3.352 (4)	163

Symmetry codes: (i) −*x*+1, −*y*+1, −*z*; (ii) *x*+1, *y*, *z*.

## References

[bb1] Brandenburg, K. (2006). *DIAMOND* Crystal Impact GbR, Bonn, Germany.

[bb2] Cunico, W., Capri, L. R., Gomes, C. R. B., Sizilio, R. H. & Wardell, S. M. S. V. (2006). *Synthesis*, pp. 3405–3408.

[bb3] Cunico, W., Gomes, C. R. B., Ferreira, M. L. G., Capri, L. R., Soares, M. & Wardell, S. M. S. V. (2007). *Tetrahedron Lett.* **48**, 6217–6220.

[bb4] Cunico, W., Gomes, C. R. B. & Vellasco, W. T. Jr (2008*a*). *Mini-Rev. Org. Chem* **5**, 336–344.

[bb5] Cunico, W., Vellasco, W. T. Jr, Moreth, M. & Gomes, C. R. B. (2008*b*). *Lett. Org. Chem* **5**, 349–352.

[bb6] Farrugia, L. J. (1997). *J. Appl. Cryst.* **30**, 565.

[bb7] Gomes, C. R. B., Moreth, M., Facchinetti, V., de Souza, M. V. N., Vellasco Junior, W. T., Lourenço, M. C. S. & Cunico, W. (2010). *Lett. Drug Des. Discov* **7**, 353–358.

[bb8] Hooft, R. W. W. (1998). *COLLECT* Nonius BV, Delft, The Netherlands.

[bb9] Kavitha, C. V., Nanjunda Swamy, B. S., Mantelingu, K., Doreswamy, S., Sridhar, M. A., Prasad, J. S. & Rangappa, K. S. (2006). *Bioorg. Med. Chem.* **14**, 2290. 10.1016/j.bmc.2005.11.017 16338140

[bb10] Neuenfeldt, P. D., Drawanz, B. B., Cunico, W., Tiekink, E. R. T., Wardell, J. L. & Wardell, S. M. S. V. (2009). *Acta Cryst.* E**65**, o3190–o3191. 10.1107/S1600536809049460 PMC2971766 21578902

[bb11] Neuenfeldt, P. D., Duval, A. R., Drawanz, B. B., Rosales, P. F., Gomes, C. R. B., Pereira, C. M. P. & Cunico, W. (2011). *Ultrason. Sonochem.* **18**, 65–67. 10.1016/j.ultsonch.2010.07.008 20724206

[bb12] Otwinowski, Z. & Minor, W. (1997). *Methods in Enzymology*, Vol. 276, *Macromolecular Crystallography*, Part A, edited by C. W. Carter Jr & R. M. Sweet, pp. 307–326. New York: Academic Press.

[bb13] Rao, A., Chimirri, A., Ferro, S., Monforte, A. M., Monforte, P. & Maria Zappalà, M. (2004). *Arkivoc*, pp. 147–155

[bb14] Ravichandran, V., Prashantha Kumar, B. R., Sankar, S. & Agrawal, R. K. (2009). *Eur. J. Med. Chem.* **44**, 1180–1187. 10.1016/j.ejmech.2008.05.036 18687505

[bb15] Rawal, R. K., Tripathi, R., Katti, S. B., Pannecouque, C. & De Clercq, E. (2008). *Eur. J. Med. Chem.* **43**, 2800–2806. 10.1016/j.ejmech.2007.12.015 18242784

[bb16] Sharma, S., Singh, T., Mittal, R., Saxena, K. K., Srivastava, V. K. & Kumar, A. (2006). *Arch. Pharm. Chem. Life Sci* **339**, 145–152. 10.1002/ardp.200500215 16528795

[bb17] Sheldrick, G. M. (2007). *SADABS* Bruker AXS Inc., Madison, Wisconsin, USA.

[bb18] Sheldrick, G. M. (2008). *Acta Cryst.* A**64**, 112–122. 10.1107/S0108767307043930 18156677

[bb19] Solomon, V. R., Haq, W., Srivastava, K., Puri, S. K. & Katti, S. B. (2007). *J. Med. Chem.* **50**, 394–398. 10.1021/jm061002i 17228883

[bb20] Westrip, S. P. (2010). *J. Appl. Cryst.* **43**, 920–925.

